# Maternal bleeding complications following early versus delayed umbilical cord clamping in multiple pregnancies

**DOI:** 10.1186/s12884-018-1781-6

**Published:** 2018-05-04

**Authors:** Chayatat Ruangkit, Matthew Leon, Kasim Hassen, Katherine Baker, Debra Poeltler, Anup Katheria

**Affiliations:** 1 Chakri Naruebodindra Medical Institute, Faculty of Medicine Ramathibodi Hospital, Mahidol University, Samut Prakan, Thailand; 2 Neonatal Research Institute at Sharp Mary Birch Hospital for Women and Newborns, 8555 Aero Dr., Suite 104, San Diego, CA 92123 USA

**Keywords:** Delayed umbilical cord clamping, Multiple pregnancy, Postpartum hemorrhage

## Abstract

**Background:**

In 2015, the American Academy of Pediatrics recommended delayed umbilical cord clamping for at least 30–60 s for all infants. However, there is limited data regarding the maternal safety of delayed cord clamping in multiple pregnancies. We aimed to compare the maternal bleeding complications following early cord clamping (ECC) versus of delayed cord clamping (DCC) in multiple pregnancies.

**Methods:**

A retrospective cohort study of pregnant women with multiples who delivered live-born infants at Sharp Healthcare Hospitals in San Diego, CA, USA during January 1st, 2016 – September 30th, 2017. Bleeding complications of 295 women who underwent ECC (less than 30 s) were compared with 154 women who underwent DCC (more than 30 s). ECC or DCC was performed according to individual obstetrician discretion.

**Results:**

Four hundred forty-nine women with multiple pregnancies (*N* = 910 infants) were included in the study. 252 (85.4%) women underwent cesarean section in ECC group vs. 99 (64.3%) in DCC group. 58 (19.7%) women delivered monochorionic twins in ECC group vs. 32 (20.8%) women in DCC group. There was no increase in maternal estimate blood loss when DCC was performed comparing to ECC. There were no differences in operative time, post-delivery decrease in hematocrits, rates of postpartum hemorrhage, bleeding complications, maternal blood transfusions and therapeutic hysterectomy between the two groups.

**Conclusions:**

No differences in maternal bleeding complications were found with DCC in multiple pregnancies compared to ECC. Delayed cord clamping can be done safely in multiple pregnancies without any increased maternal risk.

## Background

Evidence from multiple randomized controlled trials and metanalysis suggest that delayed umbilical cord clamping results in significant health benefits for term and preterm infants [1, 2]. Maintaining placenta-to-infant circulation during delayed clamping and cutting the umbilical cord after the onset of ventilation provides a more physiologic transition from fetal to neonatal life [3]. In term infants, delayed cord clamping (DCC) increases hemoglobin at birth, improves iron storage and decreases iron deficiency anemia during the first year of life when compared to early cord clamping (ECC) [2]. These effects have translated into improved neurodevelopmental outcomes at 4 years of age [4]. In preterm infants, DCC has been shown to reduce mortality [5]. Despite these neonatal benefits, there are limited data on maternal outcomes. While previous systematic reviews have reported no association between DCC and maternal risk of postpartum hemorrhage, blood loss at delivery, or need for blood transfusion [2] they have only included vaginal singleton births [6–12].

Multiple pregnancy is associated with a higher risk of maternal and neonatal complications compared to singleton pregnancy [13]. Multiple pregnancy is known to be risk factors for maternal bleeding complications [14]. Moreover, when combined with the operative delivery that is frequently performed in this patient population, the risk for bleeding complication is expected to be even higher. Our institution holds theoretical concerns regarding performing DCC in this patient population that DCC can significantly increase the duration of labor due to the delayed time spent with multiple infants which could result in increased blood loss from operation site as well as may precipitate uterine atony.

The lack of sufficient evidence in the literature in multiple gestations leads to variation in obstetric practice in this patient population. Different cord management techniques were used in previous clinical trials in this patient population including ECC, DCC, or umbilical cord milking (UCM) [15, 16]. In our institution, like most places, the decision to perform any of these procedures is based on obstetrics provider’s discretion.

We, therefore, sought to evaluate the effect of DCC in multiple pregnancies at our institution. We hypothesized that DCC in multiple pregnancies increased the risk of maternal blood loss and bleeding complication when comparing to ECC.

## Methods

We performed a retrospective cohort study by reviewing electronic medical records of all women with multiple pregnancies who delivered live-born infants at Sharp Healthcare Hospitals in San Diego, California from January 1st, 2016 to September 30th, 2017. All multiple deliveries in Sharp Healthcare Hospitals that provided obstetrics service, including Sharp Mary Birch Hospital for Women & Newborns, Sharp Grossmont Hospital, and Sharp Chula Vista Medical Center, were reviewed. Since no written protocol is available regarding umbilical cord management technique in multiple pregnancies in our institution, the umbilical cord was managed according to individual obstetrician preference in each hospital. The information on umbilical cord management technique for each delivery was extracted from the delivery record that documented the duration of umbilical cord clamping and cutting. The patients were categorized into two groups; ECC, defined as a mother who received umbilical cord clamping before 30 s in all infants and DCC, defined as a mother who received umbilical cord clamping at least after 30 s in one or more infants. 30 s was selected as the cutoff point between ECC and DCC for both term and preterm deliveries in our study to be in accordance with national recommendation [17, 18]. UCM could have been performed in either group but it was not felt to increase maternal morbidity, so it was included. Only patients with incomplete information on umbilical cord management technique during delivery were excluded. This study was approved by Sharp Mary Birch Hospital for Women & Newborns Human Research Protection Office, IRB No. 1712804. Waiver of individual patient informed consent was granted.

Patient records were reviewed for baseline characteristics including age, race, gestational age, antenatal complications, mode of delivery, anesthesia method, body mass index (BMI) at the time of delivery, number of infants, total fetal (infants) weight, and placental pathology. Outcomes of interest including estimated blood loss (EBL), post-delivery decrease in maternal hemoglobin and hematocrit, operative time (as defined by start of skin incision to complete skin closure), post-partum hemorrhage (PPH) defined as EBL > 500 mL for vaginal delivery or EBL > 1000 mL for a cesarean delivery, etiology of bleeding complications (e.g., uterine atony, placental abruption, or uterine rupture), maternal blood transfusion and therapeutic hysterectomy were reviewed. Infants mortality before hospital discharge were recorded.

### Statistics

Univariate analyses were performed to identify significant differences between the groups. Student’s t-test were used for parametric continuous variables and results were presented as mean ± standard deviation; SD. Mann-Whitney U test were used for non-parametric continuous variables. Results were specified accordingly wherever it is used and presented as median (interquartile ranges). Pearson’s Chi-square tests or Fisher Exact tests were used for categorical variables and results were presented as total number (%). A *p*-value < 0.05 was considered statistically significant. SPSS (version 23, IBM, Chicago, IL) was used for all statistical analysis.

## Results

**Fig. 1:**
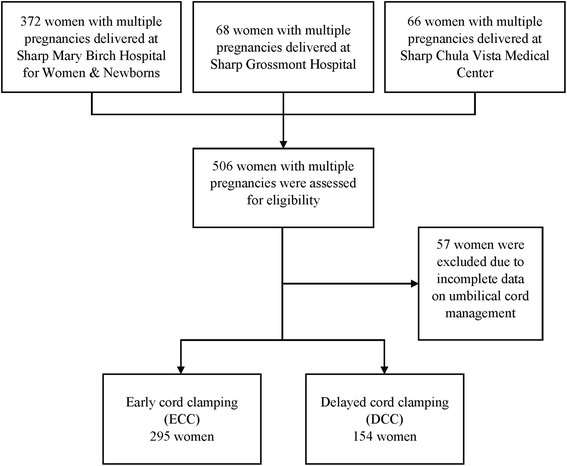
Flow chart of patients in the study

During the study period, 506 women with multiple pregnancies delivered 910 live-born infants in Sharp Healthcare Hospitals (372 women at Sharp Mary Birch Hospital for Women & Newborns, 68 women at Sharp Grossmont Hospital, 66 women at Sharp Chula Vista Medical Center). Fifty-seven women had incomplete information on umbilical cord management during the delivery and were excluded from the study. The medical records of 449 women with complete delivery information were reviewed, and the patients were categorized into two groups as previously described. Figure 1, the lowest gestational age of the women in the study was 22 + 0 weeks, and the highest gestational age was 40 + 1 weeks. Two hundred ninety-five women in ECC group gave birth to 600 infants, and 154 women in DCC group gave birth to 310 infants. One hundred seventy-six women (59.7%) in ECC group and 113 women (73.4%) in DCC group delivered before 37 weeks. DCC more than 30 s was performed successfully in all infants at the delivery in 122 women (79.2%) in DCC group and in the remaining 32 women (20.8%), DCC was successfully performed only with one of the infants. During the study period, UCM was performed concurrently in 336 infants (36.9%), and 901 infants (99.0%) survived to hospital discharge.

Patients demographic and baseline clinical characteristics were comparable between the two groups. However, the mean gestational age and the mean total fetal weight in the ECC group were statistically higher than the DCC group. Patients in ECC group were more likely to deliver by cesarean section and less likely to be in active labor at the time of delivery. There were 10 triplets (8 in ECC group and 2 in DCC) and 1 quadruplets (ECC group) in the cohort. There were similar rates monochorionic twins in each group (19.7% in ECC group and 20.8% in DCC group (Table 1).

**Table 1:** Maternal demographic and baseline clinical characteristics

	ECC (*N* = 295)	DCC (*N* = 154)	*p*-values
Maternal age (years)	32.2 ± 5.9	31.1 ± 6.1	0.05
Gestational age (weeks)	35.2 ± 2.9	34.5 ± 3.0	0.02
Race/Ethnicity			
White, non-Hispanic	114 (38.6)	74 (48.1)	
White, Hispanic	111 (37.6)	54 (35.1)	
Others (Black, Asian, or other race)	70 (23.7)	26 (16.8)	0.10
Multigravida	228 (77.3)	106 (68.8)	0.05
Multiparous	193 (65.4)	87 (56.5)	0.06
Monochorionic twins	58 (19.7)	32 (20.8)	0.78
3 or more multiples	9 (3.0)	2 (1.3)	0.25
Antenatal complications			
PIH	49 (16.6)	24 (15.6)	0.78
GDM	44 (14.9)	28 (18.8)	0.37
Initial hemoglobin (g/dL)	11.9 ± 1.4	11.9 ± 1.3	0.58
Initial hematocrit (%)	35.7 ± 3.8	35.7 ± 3.5	0.98
BMI (kg/m2)	32.3 ± 5.6	32.0 ± 6.4	0.64
Anesthesia method			
Local	4 (1.4)	3 (1.9)	
Epidural/Spinal	288 (97.6)	150 (97.4)	
General	3 (1.0)	1 (0.6)	0.38
In labor at the time of delivery	164 (55.6)	113 (73.4)	< 0.01
Operative delivery	253 (85.8)	99 (64.3)	< 0.01
Total fetal weight (g)	4813.3 ± 1129.7	4490.8 ± 1057.2	< 0.01

*BMI* body mass index, *GDM* Gestational diabetes mellitus, *PIH* pregnancy-induced hypertension

The results are reported as the mean ± standard deviation (SD) and frequency (percentage), and *p* < 0.05 is statistically significant

At the time of delivery, 253 (85.8%) women underwent cesarean section in ECC group vs. 99 (64.3%) in DCC group. There was a significant difference in overall maternal estimate blood loss between the two groups (809.5 ± 400.0 mL in ECC vs. 728.8 ± 312.6 mL in DCC, *p* = 0.02). No significant differences in operative time (35.3 ± 18.2 min in ECC vs. 37.4 ± 13.2 min in DCC, *p* = 0.23), post-delivery decrease in hemoglobin (2.0 ± 1.5% in ECC vs. 2.1 ± 1.6% in DCC, *p* = 0.72), post-delivery decrease in hematocrits (6.0 ± 4.6% in ECC vs. 6.2 ± 4.8% in DCC, *p* = 0.77), rates of PPH (11.9% in ECC vs 14.3% in DCC, *p* = 0.46), and maternal blood transfusion (7.8% in ECC vs. 5.8% in DCC, *p* = 0.45). No patients in ECC group underwent hysterectomy for treatment for post-partum hemorrhage. However, the procedure was performed in 3 patients in DCC group (1.0%). In multivariable linear regression model to predict EBL, operative delivery (β = 0.323, *p* <  0.001) and maternal age (β = 0.166, *p* <  0.001) significantly predicated EBL while DCC was not a significant predictor (β = − 0.008, *p* = 0.86).

Even though uterine atony was the most common cause of PPH in our study, not all patients with PPH received the diagnosis of uterine atony, and not all patients with the diagnosis of uterine atony experienced PPH. There was no significant difference in the rates of bleeding complication diagnosis between the two group (13.9% in ECC vs. 18.2% in DCC, *p* = 0.23). Uterine atony was diagnosed in 35 (11.9%) of patients in ECC group and 26 (16.9%) patients in DCC group. Other causes of bleeding complications were diagnosed in 6 (2.0%) patients in ECC group (1 placenta accreta, 1 abruptio placenta, 1 uterine rupture, 2 retain placental tissue, and 1 unspecified hemorrhage) and 2 (1.3%) patients in DCC group (1 abruptio placenta and 1 intrauterine hematoma).

When maternal outcomes were analyzed according to mode of delivery, there was no significant difference in EBL between ECC and DCC both in vaginal delivery and cesarean section. Similarly, post-delivery decrease in hemoglobin and hematocrits, PPH, bleeding complications, maternal blood transfusion, and therapeutic hysterectomy were not significantly different between ECC and DCC groups both in vaginal delivery (Table 2) and cesarean section (Table 3).

**Table 2:** Maternal outcomes in vaginal delivery

	ECC (*n* = 43)	DCC (*n* = 55)	*p*-values
EBL (mL)	581.5 ± 469.2	509.3 ± 332.7	0.40
Mean hemoglobin decrease (g/dL)^a^	2.2 ± 2.1	1.9 ± 1.6	0.63
Mean hematocrit decrease (%)^a^	6.6 ± 6.5	5.8 ± 5.1	0.67
Bleeding complications diagnosed	9 (20.9)	14 (25.5)	0.60
Uterine atony	8 (18.6)	14 (25.5)	0.42
Others	1 (2.3)	0 (0)	0.44
Postpartum hemorrhage	11 (25.6)	11 (20.0)	0.51
Blood transfusion	3 (7.0)	3 (5.5)	1.00
Hysterectomy	0 (0)	0 (0)	1.00

^a^Blood work was not routinely performed in vaginal delivery, ECC *n* = 22, DCC *n* = 23

The results are reported as the mean ± standard deviation (SD) and frequency (percentage), and *p* < 0.05 is statistically significant. *EBL* estimated blood loss

**Table 3:** Maternal outcomes in cesarean section

	ECC (*n* = 252)	DCC (*n* = 98)	*p*-values
Operation time (min)^a^	35.3 ± 18.2	37.4 ± 13.2	0.23
EBL (mL)	848.4 ± 374.3	848.5 ± 255.1	0.99
Mean hemoglobin decrease (g/dL)	2.0 ± 1.5	2.1 ± 1.6	0.55
Mean hematocrit decrease (%)	6.0 ± 4.1	6.2 ± 4.7	0.61
Bleeding complications diagnosed	32 (12.7)	14 (14.2)	0.69
Uterine atony	27 (10.7)	12 (12.2)	0.68
Others	5 (2.0)	2 (2.0)	1.00
Postpartum hemorrhage	24 (9.5)	11 (11.2)	0.63
Blood transfusion	20 (7.9)	6 (6.1)	0.56
Hysterectomy	3 (1.2)	0 (0)	0.55

^a^Operation time only recorded in cesarean section, defined by time from surgical incision to complete skin closure

The results are reported as the mean ± standard deviation (SD) and frequency (percentage), and *p* < 0.05 is statistically significant. *EBL* estimated blood loss

## Discussion

Contrary to our hypothesis, we did not find an increased rate of maternal blood loss and bleeding complications when DCC was performed in multiple pregnancies compared to ECC. Despite the theoretical concerns of increased risk of maternal blood loss secondary to increase time spent performing DCC in multiple infants which may result in delayed hysterotomy closure as well as may precipitate uterine atony, no significant increase in morbidity was found in terms of EBL, post-cesarean decreases in maternal hemoglobin and hematocrit, operative time, rate of PPH, diagnosis of other bleeding complication, maternal blood transfusion or therapeutic hysterectomy between the two groups. While overall mean EBL was higher in the ECC group, this is most likely unrelated to umbilical cord management technique. The higher EBL in ECC group most likely can be explained by significantly higher cesarean section rate in ECC group comparing to DCC group. In our study, patients who underwent cesarean section, on average, tend to lose more blood than patients who undergo vaginal delivery. Therefore, increase in cesarean section rate resulted in EBL increases in ECC group. This association was confirmed in the multivariable linear regression model. Moreover, when the data was analyzed by mode of delivery, there was no significant difference in EBL between ECC and DCC both in vaginal delivery and cesarean section.

The absence of adverse maternal outcomes when DCC was performed in multiple pregnancies could be due to a relatively short time in performing DCC compared to the entire time spent during the delivery procedure. Many obstetricians in our group are delivering the second or third infant even before the first infant has his or her cord clamped, minimizing the total length of delay. In addition, it is also possible that obstetricians may be more attentive to bleeding as a major complication when performing DCC in multiples and they may have acted to maintain hemostasis during the procedure.

As previously mentioned the rate of cesarean section was higher in ECC group compared to DCC group in our study. This is most likely secondary to the theoretical risk and logistic difficulty of performing the DCC procedure in operative delivery, so the procedure was less likely to be performed during cesarean section. The higher rate of cesarean section in ECC may also be associated with multiple pregnancies with advanced gestational age that elective caesarian section was selected as a delivery method. The higher mean total fetal weight in ECC group also support this association. An alternative explanation for the difference in maternal baseline characteristics given the earlier recommendations for DCC only in premature infants, obstetrician and neonatologists attending deliveries may have increased preference for DCC with the lower gestational ages. This may have resulted in lower average gestational age and total fetal weight in DCC group. The higher rate of the patients who were in active labor at the time of delivery in DCC group and the lower rate of operative delivery are possibly due to more preterm labor in this group as an indication for the delivery.

The overall rate of PPH in multiple pregnancies during the study period in our institution was 12.7% (11.8% in ECC and 14.4% in DCC). Our numbers were comparable to those previously reported in the literature which ranges from 4 to 24% in multiple pregnancies [19–24]. It is unclear whether DCC technique was performed in any patients of those reports, however as the number of PPH in our study falls within ranges without significant difference between both ECC and DCC methods, we are quite reassured that DCC has been performed safely in multiple pregnancies in our institution.

The fact that there were similar rates of monochorionic twins in both groups (19.7% in ECC group and 20.9% in DCC group) is worth noting. There has been a theoretical concern regarding acute placenta-fetal transfusion that may occur during the delivery of monochorionic twins [25]. This is possibly the reasons why most DCC trial excluded monochorionic twins from their study. Despite this concern, our data indicate that DCC was performed routinely in these infants by many obstetric providers in our institution as well as in other institution without any significant detrimental infant outcomes [26].

To the best of our knowledge, our study is the largest cohort to report maternal outcomes following different umbilical cord management techniques in multiple pregnancies. Previous trials on DCC have primarily excluded women (and infants) with multiple pregnancies. Many trials on DCC, however, included women with multiple pregnancies, but the focus was to evaluate and report only on infant outcomes with insufficient maternal safety outcomes [15, 16, 27]. In 2016, Kuo et al. evaluated maternal outcomes before and after implementation of an institutional DCC protocol which included singleton and twin pregnancies and found no increase in the risk of excessive maternal bleeding or other adverse maternal outcomes, but the number of women with multiples pregnancies in the study was too small to perform any subgroup analyses [28]. Our study has large enough numbers to increase our certainty that there would be minimal maternal risk with DCC in multiple pregnancies. Many DCC trials previously reported EBL or rates of PPH which can be subjective and inaccurate [29]; therefore, we also collected data on hemoglobin and hematocrit values before and after the delivery to provide objective and relatively precise information on the degree of maternal blood loss. However, we acknowledge this is a retrospective dataset which has several limitations and still would need to be validated in a prospective randomized controlled trial.

UCM was performed concurrently during the delivery to increase placental transfusion in more than one-third of the infants. Since UCM would not affect maternal outcomes, we did not include UCM in our analysis. The data on the benefits of UCM has been previously reported, and this was outside of the intent and scope of our study [15, 30].

Due to the recent American Academy of Pediatrics and The American College of Obstetricians and Gynecologists recommending universal implementation of delayed umbilical cord clamping for at least 30–60 s for vigorous infants [17, 18], the number of obstetrician performing DCC has increased. However, it is unclear whether multiples and cesarean section delivery fall into this category. High-risk patients such as those who deliver by operative delivery or multiple pregnancies should be prospectively studied in a well-controlled, adequate- powered, randomized controlled trial. Moreover, not only infant outcomes but also maternal health outcomes should be evaluated before DCC can be generalized to those patient populations. Although this study provides more evidence to support that DCC can be performed in multiple pregnancies without detrimental maternal outcomes when compared to ECC, other technique to facilitate placental transfusion that may be more efficient, practical and timely, such as UCM, should be investigated in future clinical trials.

## Conclusions

In our study, no significant difference in maternal bleeding complications was found when DCC more than 30 s were performed in multiple pregnancies compared to ECC.

## Abbreviations

BMI: Body mass index
DCC: Delayed cord clamping
EBL: Estimated blood loss
ECC: Early cord clamping
GDM: Gestational diabetes mellitus
PIH: Pregnancy-induced hypertension
PPH: Postpartum hemorrhage
UCM: Umbilical cord milking

## References

[1] Rabe H, Diaz-rossello JL, Duley L, Dowswell T (2012). Effect of timing of umbilical cord clamping and other strategies to influence placental transfusion at preterm birth on maternal and infant outcomes. Cochrane Database Syst Rev.

[2] Mcdonald SJ, Middleton P, Dowswell T, Morris PS (2013). Effect of timing of umbilical cord clamping of term infants on maternal and neonatal outcomes. Cochrane Database Syst Rev.

[3] Bhatt S, Alison BJ, Wallace EM (2013). Delaying cord clamping until ventilation onset improves cardiovascular function at birth in preterm lambs. J Physiol.

[4] Andersson O, Lindquist B, Lindgren M, Stjernqvist K, Domellöf M, Hellström-westas L (2015). Effect of delayed cord clamping on neurodevelopment at 4 years of age: a randomized clinical trial. JAMA Pediatr.

[5] Fogarty M, Osborn DA, Askie L, et al. Delayed vs early umbilical cord clamping for preterm infants: a systematic review and meta-analysis. Am J Obstet Gynecol. 2017; [Epub ahead of print] 10.1016/j.ajog.2017.10.231 29097178

[6] Oxford Midwives Research Group (1991). A study of the relationship between the delivery to cord clamping interval and the time of cord separation. Midwifery.

[7] McDonald SJ (1996). Management in the third stage of labour [dissertation].

[8] Geethanath RM, Ramji S, Thirupuram S, Rao YN (1997). Effect of timing of cord clamping on the iron status of infants at 3 months. Indian Pediatr.

[9] Ceriani Cernadas JM, Carroli G, Pellegrini L (2006). The effect of timing of cord clamping on neonatal venous hematocrit values and clinical outcome at term: a randomized, controlled trial. Pediatrics.

[10] Chaparro CM, Neufeld LM, Tena Alavez G (2006). Effect of timing of umbilical cord clamping on iron status in Mexican infants: a randomised controlled trial. Lancet.

[11] Van Rheenen P, De Moor L, Eschbach S, De Grooth H, Brabin B (2007). Delayed cord clamping and haemoglobin levels in infancy: a randomised controlled trial in term babies. Tropical Med Int Health.

[12] Andersson O, Hellström-westas L, Andersson D, Domellöf M (2011). Effect of delayed versus early umbilical cord clamping on neonatal outcomes and iron status at 4 months: a randomised controlled trial. BMJ.

[13] Su RN, Zhu WW, Wei YM (2015). Maternal and neonatal outcomes in multiple pregnancy: a multicentre study in the Beijing population. Chronic Dis Transl Med..

[14] Nyfløt LT, Sandven I, Stray-Pedersen B, et al. Risk factors for severe postpartum hemorrhage: a case-control study. BMC Pregnancy and Childbirth. 2017;17:17. 10.1186/s12884-016-1217-0 PMC5223545 28068990

[15] Katheria AC, Truong G, Cousins L, Oshiro B, Finer NN (2015). Umbilical cord milking versus delayed cord clamping in preterm infants. Pediatrics.

[16] Tarnow-mordi W, Morris J, Kirby A, et al. Delayed versus immediate cord clamping in preterm infants. N Engl J Med. 2017; [Epub ahead of print] 10.1056/NEJMoa1711281 29081267

[17] Weiner G, Zaichkin J, Kattwinkel J (2016). Textbook of neonatal resuscitation.

[18] Committee on Obstetric Practice (2017). Committee Opinion No. 684: delayed umbilical cord clamping after birth. Obstet Gynecol.

[19] Santana DS, Cecatti JG, Surita FG (2016). Twin pregnancy and severe maternal outcomes: the World Health Organization multicountry survey on maternal and newborn health. Obstet Gynecol.

[20] Conde-agudelo A, Belizán JM, Lindmark G (2000). Maternal morbidity and mortality associated with multiple gestations. Obstet Gynecol.

[21] Qazi G (2011). Obstetric and perinatal outcome of multiple pregnancy. J Coll Physicians Surg Pak.

[22] Su RN, Zhu WW, Wei YM (2015). Maternal and neonatal outcomes in multiple pregnancy: a multicentre study in the Beijing population. Chronic Dis Transl Med.

[23] Rather S, Habib R, Sharma P (2014). Studying pregnancy outcome in twin gestation in developing world. IOSR Journal of Dental and Medical Sciences.

[24] Suzuki S, Kikuchi F, Ouchi N (2007). Risk factors for postpartum hemorrhage after vaginal delivery of twins. J Nippon Med Sch.

[25] Lopriore E, Sueters M, Middeldorp JM, Vandenbussche FP, Walther FJ (2005). Haemoglobin differences at birth in monochorionic twins without chronic twin-to-twin transfusion syndrome. Prenat Diagn.

[26] Ruangkit C, Moroney V, Viswanathan S, Bhola M (2015). Safety and efficacy of delayed umbilical cord clamping in multiple and singleton premature infants - a quality improvement study. J Neonatal Perinatal Med.

[27] Kugelman A, Borenstein-levin L, Riskin A (2007). Immediate versus delayed umbilical cord clamping in premature neonates born < 35 weeks: a prospective, randomized, controlled study. Am J Perinatol.

[28] Kuo K, Gokhale P, Hackney DN, Ruangkit C, Bhola M, March M (2018). Maternal outcomes following the initiation of an institutional delayed cord clamping protocol: an observational case-control study. J Matern Fetal Neonatal Med.

[29] Hancock A, Weeks AD, Lavender DT (2015). Is accurate and reliable blood loss estimation the “crucial step” in early detection of postpartum haemorrhage: an integrative review of the literature. BMC Pregnancy and Childbirth.

[30] Katheria A, Mercer J, Brown M, et al. Umbilical cord milking at birth for term newborns with acidosis: neonatal outcomes. J Perinatol. 2017; [Epub ahead of print] 10.1038/s41372-017-0011-9 29234144

